# ModEx: a general purpose computer model exploration system

**DOI:** 10.3389/fbinf.2023.1153800

**Published:** 2023-05-25

**Authors:** Hamid Younesy, Joseph Pober, Torsten Möller, Mohammad M. Karimi

**Affiliations:** ^1^ School of Computing Science, Simon Fraser University, Burnaby, BC, Canada; ^2^ Imperial College London, London, United Kingdom; ^3^ Research Network Data Science and Faculty of Computer Science, University of Vienna, Vienna, Austria; ^4^ Comprehensive Cancer Centre, School of Cancer and Pharmaceutical Sciences, Faculty of Life Sciences and Medicine, King's College London, London, United Kingdom

**Keywords:** visualization, computer model exploration, parameter space analysis, bioinformatics, visual analysis tools, clustering, differential gene expression, neural networks

## Abstract

We present a general purpose visual analysis system that can be used for exploring parameters of a variety of computer models. Our proposed system offers key components of a visual parameter analysis framework including parameter sampling, deriving output summaries, and an exploration interface. It also provides an API for rapid development of parameter space exploration solutions as well as the flexibility to support custom workflows for different application domains. We evaluate the effectiveness of our system by demonstrating it in three domains: data mining, machine learning and specific application in bioinformatics.

## 1 Introduction

Many complex computer models require users to provide several parameters to tune the output based on the specific computational needs. Such models appear in a variety of modeling approaches, such as data science (regression, classification, and clustering) but also computational science (numerical modeling). Today, there is almost no field of science untouched by the application of such modeling techniques. In many applications, fully automated optimization is not possible due to the trade-offs between the often contradicting objectives. A classical example is the trade-off between precision and recall for classification problems, where a higher value of one leads to a lower value for the other. This requires a domain expert to inspect different models (or results of the modeling process) to make an informed decision. Manual trial and error of running models with different parameter settings is tedious and usually requires many iterations involving guess-work and luck. In addition, the required effort for trying different parameters increases exponentially with the number of the parameters of the model. This not only makes the exploration of the parameter space extremely inefficient, it also prevents a global understanding of the model capabilities.

To address this, several systems and workflows have been developed to automate the process. These solutions run the model with different combinations of parameter values sampled from parameter space. This is done in a preprocessing step and the results are collected. Then a visual exploration interface is used to systematically explore the parameter space and further analyze the results. Many of the systems that support these types of *“visual parameter space analysis”* (*vPSA* Sedlmair et al. (2014)) techniques are built for various modeling approaches and applications domains such as simulation Potter et al. (2009); Waser et al. (2010); Bergner et al. (2013); Luboschik et al. (2014); Wang et al. (2017), image analysis Torsney-Weir et al. (2011); Pretorius et al. (2015); Fröhler et al. (2016); Teodoro et al. (2017), data mining Padua et al. (2014); Kwon et al. (2018), industrial decision making Afzal et al. (2011); Booshehrian et al. (2012); Pajer et al. (2017) and bioinformatics Hess et al. (2014). These solutions are custom-made and developed for specific application domains. The cost of building these tools is usually high and often involves several months of development work. As a result, parameter exploration solutions lack a wide-spread adoption for many computer models that can benefit from the approach.

In this paper we propose ModEx, a general purpose system that facilitates visual parameter analysis for a wide variety of computer models. Our system offers key components of a visual parameter analysis framework including parameter sampling, deriving output summaries and an exploration interface, which can work with a wide range of tools and libraries minimizing the required setup time. It also provides a flexible API for rapid development of parameter space exploration solutions for custom application domains. We demonstrate this flexibility and effectiveness in three application domains: data mining, machine learning, and bioinformatics.

As part of our vPSA system introduced in Section 4, we make three primary contributions. First, we present a novel method of creating user interfaces suitable for specifying the relevant parameter space and parameter sampling. These interfaces are created automatically and seamlessly for computer models wrapped into modules called apps using a parameter description API. Second, we present a generic approach to handle derivations of outputs and show how it can be extended for custom application domains. Our third primary contribution is the design and implementation of a visual exploration interface to allow analyzing the results of parameter sampling, either to find “right” parameters for a specific problem (for model users), or to get a deeper understanding of a computer model’s inner workings (for model developers). The user interface is designed to be flexible and to allow creating custom workflows by linking either existing computational modules or creating new ones.

Our secondary contribution is an evaluation of our system in different application domains. We present three case studies: First, we consider the problem of clustering and demonstrate how ModEx can be used to quickly recreate the main functionality of similar state-of-the art tools for comparing different cluster algorithms. In our second case study, we use ModEx to analyze two widely used bioinformatics methods. In our third case study, we study the problem of hyperparameter tuning for neural networks and use a popular educational neural network application as our model for ModEx.

## 2 Related work

Sedlmair et al. (2014) introduced the conceptual framework for visual parameter space analysis (vPSA). They identified a dataflow model with the essential components of *the model*, a *surrogate model* as well as the *derive* step. Their work was based on 21 papers from the visualization community. This was the main inspiration of our work and we followed their guidelines. We are supporting the *model* component as well as the *derive* component. However, currently we do not support the *surrogate* model—only 5 of the 21 papers analyzed by Sedlmair et al. used such a component. Still, such a component is of importance but is left as future work for now. Since the publication of Sedlmair et al. (2014), the design of parameter exploration systems has continued. We will report on the state-of-the-art for the specific application domains we do consider in this paper. We will not report vPSA systems in other domains not directly relevant to this paper.

### 2.1 Applications in machine learning

The terms *Machine Learning* as well as *Data Mining* are often considered to be rather broad terms encompassing techniques in regression analysis, classification analysis, clustering, outlier detection, and dimensionality reduction. For the purpose of this paper, however, we consider the focus and contributions of machine learning to be mainly in the area of classification analysis and the contribution and focus of data mining to be mainly in the area of clustering analysis.

As far as classification is concerned, TreePOD by Mühlbacher et al. Mühlbacher et al. (2018) supports a user in understanding the trade-offs of various decision trees. Through a parameter sampling approach, a large number of decision trees are built. They are then evaluated based on accuracy in addition to aspects such as ease of understanding by decision makers. The user is then able to inspect relevant trees that are on the Pareto Frontier of such trade-offs.

One of the most promising approaches for machine learning today are deep neural networks Goodfellow et al. (2016). There has been a lot of attention paid to the analysis of particular deep neural networks. One of the hardest challenges considered today is the understanding of a proper network architecture, or the determination of proper hyperparameters. Recent surveys by Liu et al. (2017) and Hohman et al. (2018) focus on visual analytics approaches, however, to our best knowledge there is no generic vPSA framework solution addressing this problem.

### 2.2 Applications in data mining

In data mining and particularly clustering, Kwon et al. (2018) presents a comprehensive discussion of clustering methods and visualization systems for cluster analysis and discusses several categories of systems. One group are “visual analytics systems that employ clustering as a part of high dimensional data analysis” which include Hierarchical Clustering Explorer (HCE) Seo and Shneiderman (2002), VISTA Chen and Liu (2004), and DICON Cao et al. (2011). A second group are those which “allow users to provide feedback on clustering results so that the next run applies their inputs” such as desJardins et al. (2007), iVisClustering Lee et al. (2012), Cluster Sculptor Bruneau et al. (2015), Boudjeloud-Assala et al. (2016), and Clusterix Maguire et al. (2016). A third group are those which “allow users to generate and compare multiple clustering results with respect to their quality”. Example research falling in this group are Turkay et al. (2011) and XCluSim L’Yi et al. (2015).

### 2.3 Meta tools

Another category of systems related to ModEx are so called meta tools or visualization design environments which are developed with the intention of making the visualization creation process accessible to non-programmers. These tools provide an interactive environment with drag-and-drop interactions to allow creation of customized linked visualizations and visual analysis workflows without requiring textual programming. Among the most known tools in this category are Lyra Satyanarayan and Heer (2014), iVisDesigner Ren et al. (2014), Voyager Wongsuphasawat et al. (2016), Voyager2 Wongsuphasawat et al. (2017), iVoLVER Méndez et al. (2016), as well as commercial tools such as Tableau Stolte et al. (2002) and Keshif Yalçın et al. (2018). Most of these tools utilize a visualization grammar such as the Grammer of Graphics Wilkinson (2006), Vega Satyanarayan et al. (2014) and Vega-Lite Satyanarayan et al. (2017), or a visualization toolkit such as Protovis Bostock and Heer (2009), and D3 Bostock et al. (2011), to describe the visual appearance and interactive behaviour of the visualizations. Some also provide an interface to programming languages such as python, R, or javascript to increase expressiveness and flexibility for more advanced users.

### 2.4 Definitions

The terminology used throughout this paper, closely follows the guidelines in the vPSA conceptual framework Sedlmair et al. (2014). For clarity, we will briefly explain some of the most frequent terms used in our paper.

• *Computer model* or simply the model, is the algorithm or set of algorithms to which the vPSA system is being employed. In this paper with computer models we mean statistical models as it is common in data science or simulation models as it is common in computational science. The terminology used in the vPSA conceptual framework is *computational input-output models.*
• *Run* is one execution of the model with specific parameter values.
• *Parameter combination* is the randomly generated set of values used as *control parameters* in a single execution of the model. Different runs of the model will often use different parameter combinations.
• *Derived output* are objective measures that summarize the essential characteristics (e.g., quality) of the complex model output.

## 3 VisR system

We built our parameter exploration system as part of the VisR Younesy et al. (2015) framework (formerly called VisRseq). VisR is a visual analysis system that provides an accessible interface for non-programmers to run computational libraries implemented in the R programming language R Core Team (2019a). VisR was originally designed to address the challenges in the domain of genome sequencing (hence the suffix -seq), but has evolved toward a more generic visual analysis system.

### 3.1 VisR apps

**FIGURE 1:**
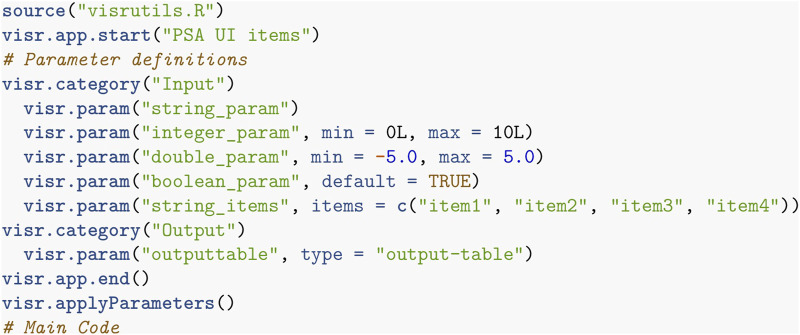
R code for an R-app in the VisR framework specifying the app’s name, the parameters, and the code to perform the desired functionality.

**FIGURE 2:**
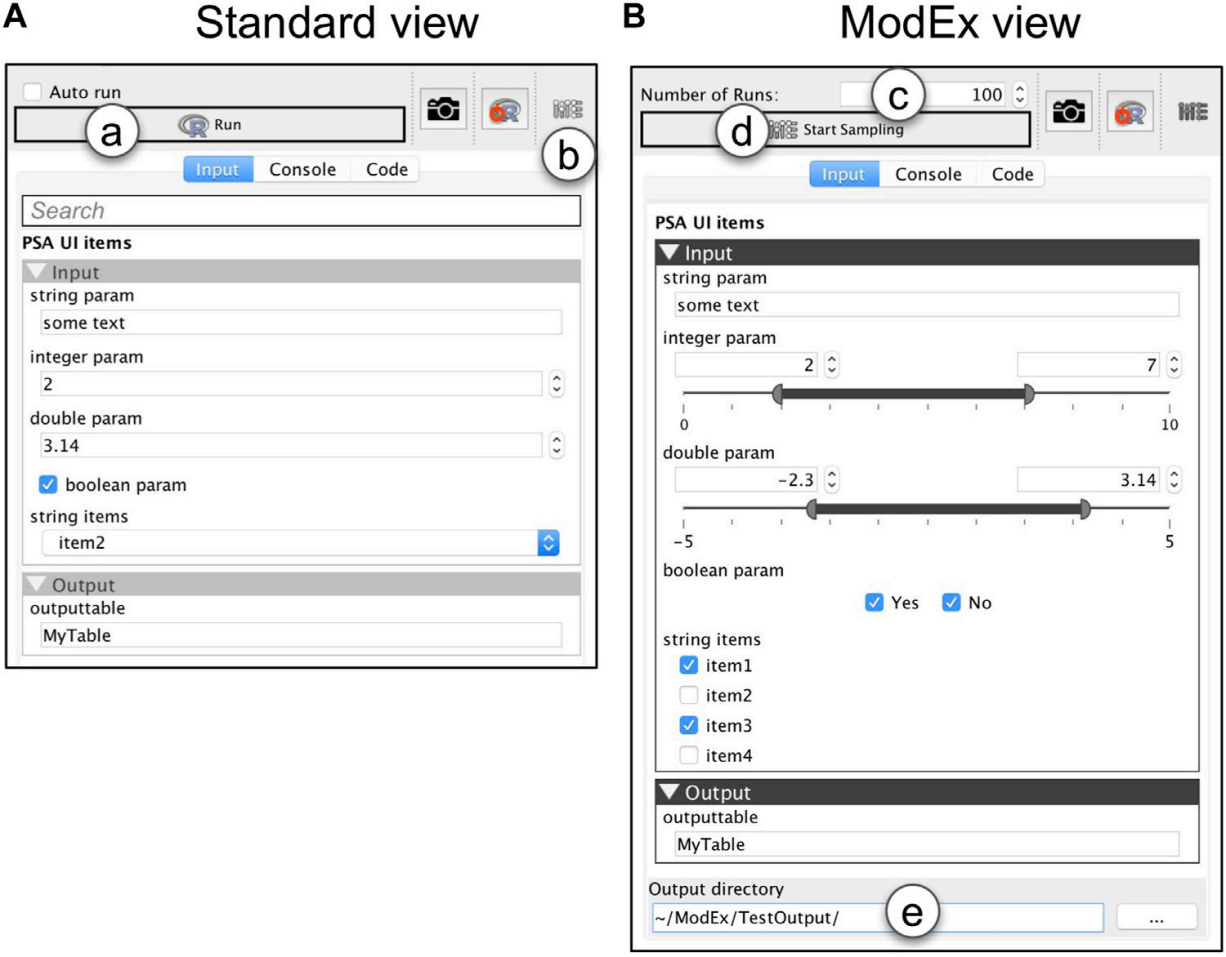
The GUI generated from the parameter specification in Figure 1: **(A)** Standard view, and **(B)** ModEx view. (a) run the R-app. (b) switch the GUI to ModEx view. (c) number of times to run the app. (d) start running the R-app for the specified number of runs. (e) directory to store the output of runs.

The functional units in VisR are called apps. A small number of the apps are *interactive apps*, are developed natively to allow interactive exploration and filtering. A larger number of the apps are developed in R and are called *R-apps*. The graphical user interface for an R-app is created automatically from a simple specification of the parameters similar to the toy example shown in Figure 1. The graphical user interface generated for the code is shown in Figure 2A. To run an app, a user drag-and-drops the app icon to the workspace to create an active window for the app. The user then specifies the input and parameters through the app’s graphical user interface. For interactive apps the app updates in real time as the user interacts with the active window or modifies the parameters. For R-apps, the user specifies the parameters and then presses the “Run” button (Figure 2A). VisR will then pass the user’s input data and parameters to the R environment, run the corresponding code and return the output results back to the workspace. The output can be images, data tables, columns appended to the input data table or files. The user can then use other apps to explore the results and link multiple apps to create more complex workflows.

### 3.2 The challenge

The rapid development API of VisR Younesy (2016) allowed us to create many (50+) R-apps in a short period of time and the usage shows a growing number of users since the release (600+ users from 30+ countries). However, even though the graphical user interface of the R-apps allowed non-programmers to utilize the computational power of the R environment, a new challenge surfaced. Most apps have several (typically between 3 and 5, but sometimes over 10) numerical or categorical control parameters. Although the parameters for each app were initialized with the default values suggested in the corresponding R package and some documentation was provided in the form of tool tips, finding the “right” parameters for each use case involved a tedious manual trial-and-error approach. Encouraged by the previous successful attempts with visual parameter exploration tools we implemented ModEx as an extension of VisR to support general visual parameter space analysis for R-apps. The next section discusses our approach.

## 4 A general purpose system for visual parameter space analysis

This section introduces our general purpose system for visual parameter exploration of computer models. We were initially focused on addressing the challenges in the bioinformatics application domain. However, through the development process we realized that most of the analysis tasks are generic and applicable in other application domains. As such ModEx has iteratively evolved to be usable in a wider variety of application domains. We will demonstrate this generality in Section 5.

### 4.1 Design goals

From the very early phases, an essential design goal was to develop ModEx such that it follows the same design principles of VisR to avoid alienating the existing user base. We also wanted to utilize the methodology of vPSA for existing R-apps without requiring significant development. In addition, we wanted the possibility of utilizing ModEx for models not implemented in R. We will elaborate our design decisions based on these goals in the remainder of this section.

### 4.2 Parameter sampling

Our first contribution is extending the existing automated GUI system so that it can be used to specify a range (or space) of parameter combinations. Figure 2A shows the generated standard (single parameter) view which is the default when a user adds an app to the workspace. In a typical scenario, a user specifies the parameters through the standard view and then clicks on the “Run” button (Figure 2A). VisR then passes the user specified parameters to the R environment, executes the app’s code, and returns the results back to the workspace for further exploration. In ModEx, a user can now click on the toolbar button shown in Figure 2B to toggle between the standard view and the ModEx view mode shown on Figure 2B. In the ModEx view mode the user interface controls are generated such that they allow specifying ranges of valid values for the parameters as follows:

• *integer/double*: integer/double lower and upper bounds for the parameter sampling range. The acceptable minimum and maximum can be specified in the app’s declaration script (see Figure 1).
• *Boolean*: two check boxes with options yes and no
• *string* with defined items: a check box for each item.
• other parameter types: such as generic string, filenames, or output parameters will not require custom made UI controls.

UI controls are initially set to the default value specified in the app’s declaration script. At that state, the value for the corresponding parameter will stay constant during the parameter sampling. This allows users to choose which parameter values are kept unchanged and which ones are sampled randomly from the specified range. Currently only random uniform sampling is implemented and exploration of other sampling methods is discussed as a possible future work in Section 8.1. The total number of parameter combinations can be modified by changing the value of “Number of Runs” shown in Figure 2C. We initially had the option to specify either the number of runs, or an end time when sampling should be stopped. However using end time turned out to be inconvenient as different computer models had significantly varying execution times which could result either in too many or too few number of runs. We instead decided to allow users to stop any time without corrupting the final output. They can also resume the runs at a later time if they later realize more samples are required. This allows partial and off-line (as opposed to real-time) support for the *simulation steering* strategy explained in the conceptual framework where the user can make adjustments while the computer model runs, for example, to change some input parameter settings.

**FIGURE 3:**
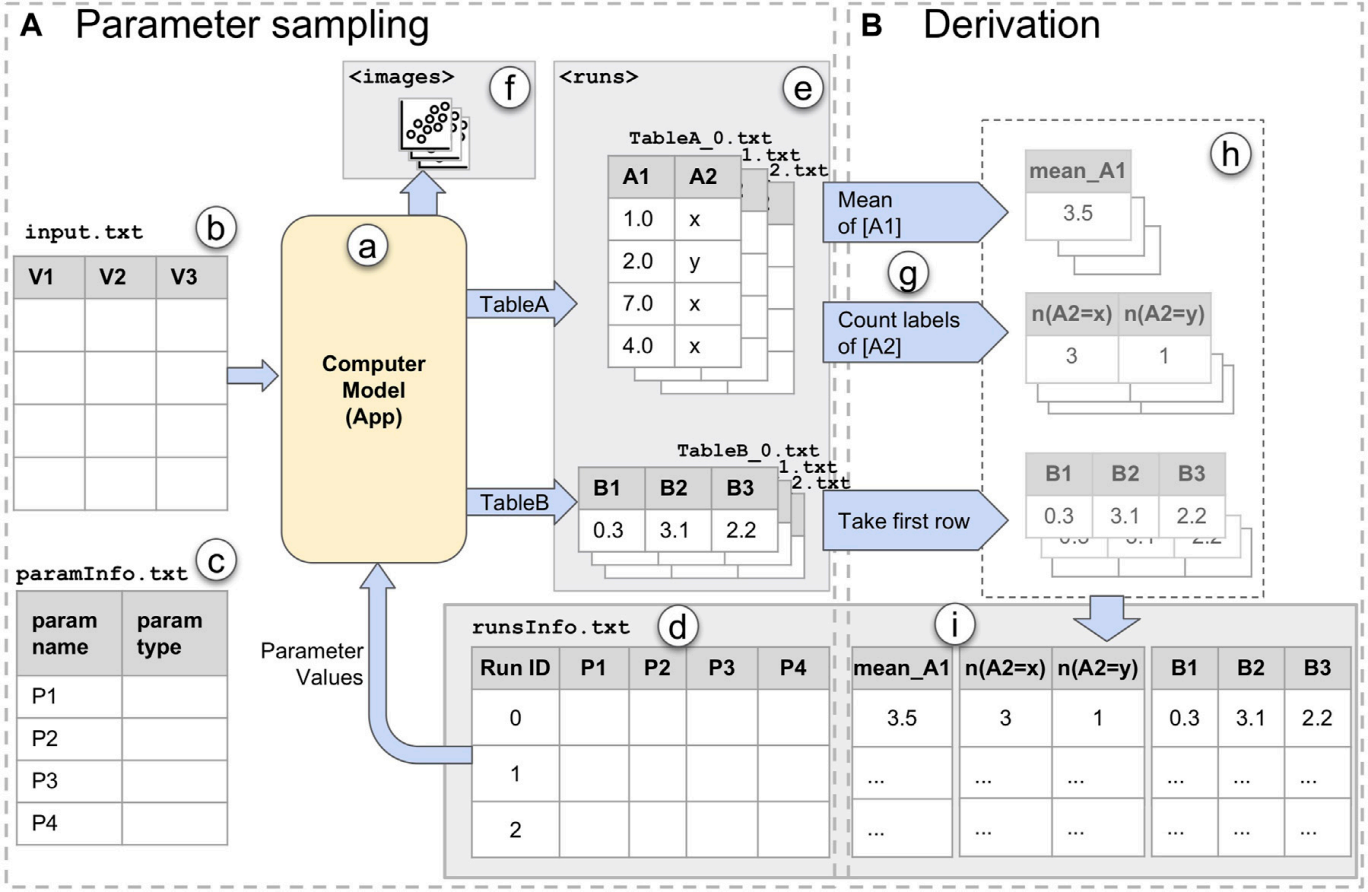
The ModEx data flow: **(A)** Parameter Sampling step; **(B)** Derivation step. Parameter sampling includes: (a) the computer model/app; (b) input data to the app; (c) parameters of the app; (d) runsInfo table containing information about each run, e.g., parameter values; (e) data outputs of the app; (f) image outputs of the app. Derivation includes: (g) derivation methods; (h) derived output (in memory); (i) derived output added to the runsInfo table.

The parameter sampling will start when a user clicks on the “Start Sampling” button (Figure 2D) and the results are stored in the user specified directory (Figure 2E). The sampling process is demonstrated in Figure 3A and includes the following:

• *input.txt*: the data table used as the app’s input, shown in Figure 3B.
• *paramInfo.txt*: a data table containing the name and type of the app’s parameters, shown in Figure 3C.
• *runsInfo.txt*: a data table with each row corresponding to a single run and columns containing the values for the parameter combination, as shown in Figure 3D. In addition to the parameter combination values, each run is also assigned with a unique integer ID used to reference the output for that run.
• *< runs>*: subdirectory containing all output from the runs, each suffixed with the run ID. For instance if the R-app outputs two data tables, TableA and TableB, the < runs > directory will contain TableA_0.txt, TableB_0.txt, TableA_1.txt, TableB_1.txt and so on, such as the example shown in Figure 3E.
• *< images>*: subdirectory containing image outputs from the runs, named as [ID].[ext], e.g., 0.png, 1.png, and so on, as shown in Figure 3F. This can be extended to include other complex objects.

This directory structure is the same for all R-apps sampled in ModEx. To use ModEx with non-R models, there are two alternatives: If the model has a command-line interface, we can create an R-app that includes the parameter specification, but utilize the R’s “system” function R Core Team (2019b) to invoke the non-R tool with the user parameters. The second alternative, which we employed in Section 7, is to perform the parameter space sampling and execution of runs outside ModEx and to collect the results in a directory structure compatible with the above.

### 4.3 ModEx app

To explore and analyze the output of the runs, we built an interactive VisR app, the *ModEx* app. Once users have launched ModEx, they have to first specify the runs’ output directory. The app will then process the directory and show the UI options for further analysis. The ModEx app provides two of the main components of a vPSA framework: computing derived output, and an interactive exploration interface. The following sections describe the design and functionality of each component.

#### 4.3.1 Derivation

To be able to effectively analyze the results of many runs of a computer model, we have to summarize its output into objective measures referred to as derived output. However, supporting the functionality to compute the derivations for a wide array of models is an extremely challenging endeavor due to the wide variety of the output types. E.g., image segmentation or clustering algorithms output a different label for each pixel/input element, classifiers output a label for each input, etc. In order to balance between flexibility and usability, our system offers a set of out-of-the-box derivation methods, as well as the means to have custom derivation methods when needed. The data flow for the derivation step is illustrated in Figure 3B. Output derivation can be performed in one or more passes. In each derivation pass, shown in Figure 3G, a user selects a derivation method as well as the output data to which the derivation should be applied. The outcome will be a vector of one or more numerical or categorical values per run, examples of which are shown in Figure 3H. These values will be collected and appended to the runsInfo table as new columns with labels generated based on the derivation method as shown in Figure 3I. The choices for out-of-the-box derivations methods are:

• *Aggregate*: includes eight derivation methods. A group of these functions (*mean*, *sum*, *median*, *min*, and *max*) are used to aggregate numerical data columns. Another group are meant for categorical output: *Number of class labels* counts the number of different class labels, *Mode of class labels* finds the mode (label occurring the most), and *Count per class label* counts the occurrence (histogram) of each different class label.
• *Comparison with ground truth*: is useful for analyzing models where a data set with ground truth output exists. For example, in a supervised classification model, the training data will have ground truth labels. This method will compare the predicted labels for each run with the ground truth labels, and output the number of matches and mismatches per label. For a detailed example usage see Supplementary Material.
• *Dimensionality Reduction*”: provides MDS, PCA, and tSNE functions. All perform dimensionality reduction on the selected output. An example usage will be discussed in Section 5.1.
• *Take first row*: will just use the first row of the selected output table. Despite its simplicity, that is actually what enables embedding custom derivation methods in computer models. It lets a computer model itself to compute any special derived output and export them as a table with a single row. For instance in Section 5.1, the clustering app computes clustering quality metrics as derived output for each run.
• *None*: skips the derivation pass altogether. This is helpful when runsInfo contains the necessary derived output already, e.g., when a different tool was used for parameter sampling and derivation, or when the derivation step had already been performed.

We will further discuss the usage of the derivation methods in our case studies in Section 5. But for now let’s go through a simple example based on the data flow in Figure 3B. The result of applying the “mean” method on the column “A1” of “TableA” generates a column “mean_A1”. In a second derivation pass, applying the “Count labels” method on the column “A2” of “TableA”, results in two new columns labeled “n(A2 = x)” and “n(A2 = y)”, corresponding to the total number of “x” or “y” values in “A2” for each run. In another derivation pass, using the “Take first row” method on “TableB” will take the first rows as-is and add the columns “B1”, “B2”, and “B3” to the runsInfo table.

**FIGURE 4:**
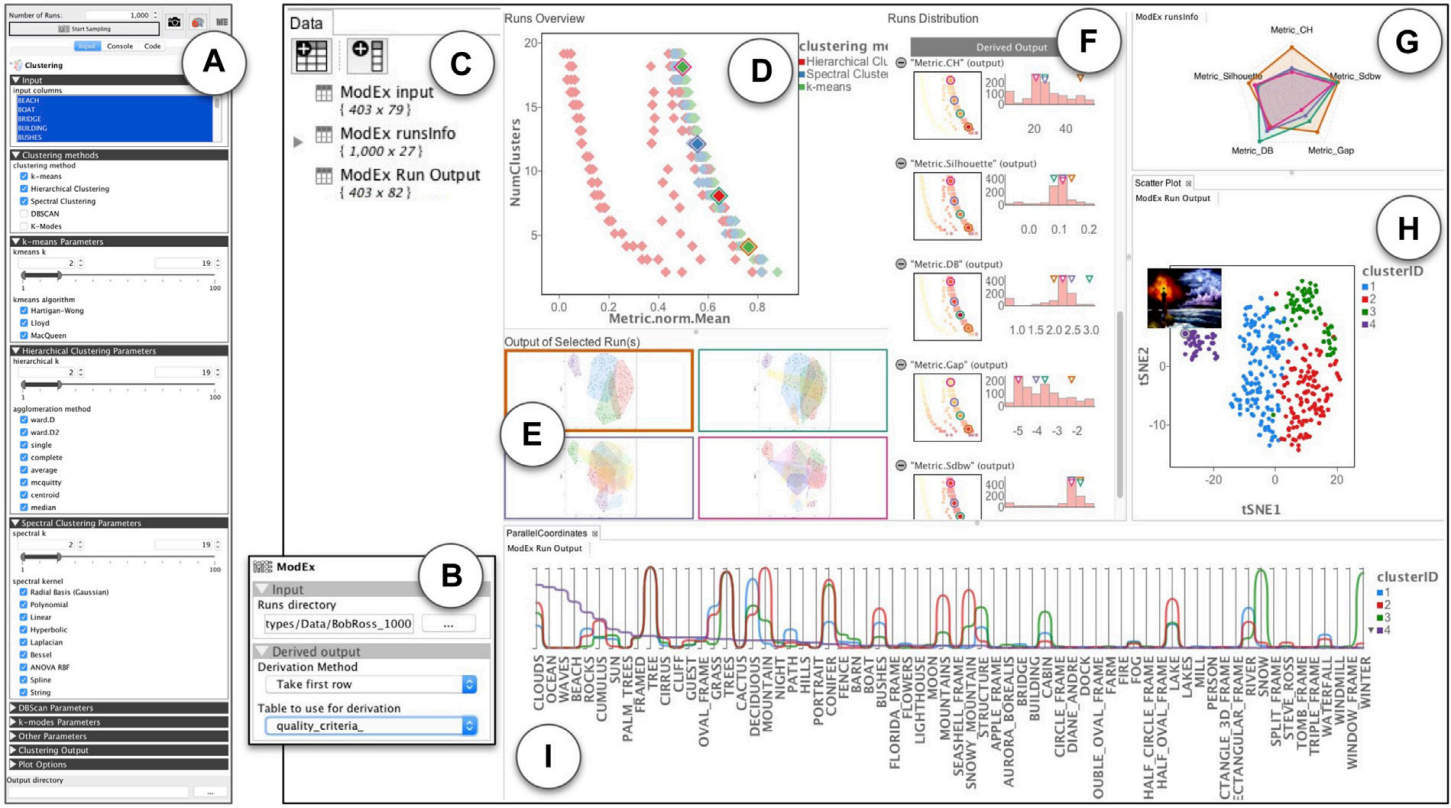
An overview of *ModEx* used for visual parameter exploration in a clustering application domain: **(A)** ModEx parameter view mode for the clustering app; **(B)** Calculating derived output; **(C–F)** The generic parameter space exploration interface; **(G–I)** Additional apps linked to the generic exploration interface to create a customized workflow; **(C)** Data view providing access to the data tables in the workspace; **(D)** *Runs Overview* showing a scatter plot of derived outputs (here the aggregated quality metric vs. number of clusters) for runs; **(E)** Image *Output of Selected Runs* highlighted in the *Runs Overview* plot. **(F)** *Runs Distribution* showing a list of histogram distributions and filters for input parameters and derived output; **(G)** Radar plot showing the quality metrics for the selected runs; **(H)** Scatter plot linked to the output of the latest selected run; **(I)** Lineplots/parallel coordinates linked to the output of the latest selected run.

Figure 4B shows the UI for the derivation step. To keep the user interface simple, each pass will only perform one derivation method on one of the output data tables. However, multiple passes can be performed to generate additional types of derived output as needed. When all passes are performed, a user can select the “Start Exploration” option so that the exploration interface is launched immediately. It is worth noting that the user interface and functionality of the derivation component is also developed as an R-app, so like all other R-apps, its UI and functionality can be easily extended or customized for future needs.

#### 4.3.2 Exploration

The core exploration interface of ModEx, shown in Figures 4C–F, allows the user to interactively explore the results of parameter space sampling. Here we will briefly introduce different views and features and will demonstrate more details through the case studies in Section 5.

The “Runs overview” in Figure 4D shows an interactive scatter plot of the runsInfo table where each data point is one of the runs of the computer model. As a user hovers the mouse pointer over a run data point, a thumbnail of the output of the run is shown as a tooltip. When user clicks on a run data point, it is selected and its image output is displayed on the “Output of Selected Run(s)” pane as shown in Figure 4E. Multiple runs can be selected by holding the shift button. The user can specify a parameter or derived output column as the scatter plot axis by clicking on the axis label, and can change the visual properties such as the point size, color map, axis scaling mode, etc. using the parameters pane (not shown in the figure). The runsInfo table as well as the output tables for the currently selected run can be accessed through the data pane Figure 4C and are updated as the user changes the currently selected run. This allows the user to chain the outputs to any of the apps available in VisR. We will see examples of this in Section 5.1.

The “Runs Distribution” view shown in Figure 4F displays a list of the parameters and derived outputs of the computer model. Each item in the list consists of a histogram (bar plot) and a smaller scale of the “Runs Overview” scatter plot. The histograms have active brushing which can be used to filter the runs with the desired values for the parameters or derived outputs. A user can click on the (−) icons beside each list item, to minimize (collapse) the plots for that list item. The values for the currently selected run(s) and the hovered run under the mouse cursor are indicated by up-side-down triangles above the histograms. The scatter plots use the same axis as the “Runs Overview”, but color the run data points based on the values of the parameter or derived output for the corresponding list item to allow a user to quickly check for any specific patterns. Selecting a list item updates the “Run Overview” plot to use the selected item for colouring the run data points. To reduce redundancy we chose to hide the legend by default and instead use the colors shown for the histogram labels. A user can however turn it back on through the scatter plot app’s parameters.

The core interface described above provides the initial means for exploratory analysis of the runs, however the main strength of ModEx comes from the ability to add other apps to the workflow to extend and customize its functionality based on the specific application domain. We will further explore this in the upcoming section.

## 5 Evaluation

To demonstrate the effectiveness of our framework on different application domains, we will go through three usage scenarios two of which are provided as Supplementary Material. First, we will look at a clustering problem and compare our results with a tool developed recently to address this problem. In the second case study provided as Supplementary Material, we will study the problem of setting hyperparameters for training of neural networks. We will use an educational neural network application as our model and show how ModEx can help find proper values for the hyperparameters. In the third case study also provided as Supplementary Material, we will look at two commonly used bioinformatics methods and report the insights gained from utilizing ModEx.

### 5.1 Case study: clustering

In our first case study, we will look at the data mining application domain and evaluate the effectiveness of ModEx by comparing it to a recently published visual parameter space analysis tool called Clustervision Kwon et al. (2018). Clustervision was developed to help with finding the right parameters for unsupervised clustering. As one of their case studies, Kwon et al. analyze the “Bob Ross Paintings” dataset created by Hickey Hickey (2014). They state that they “use this dissatisfaction by Hickey to motivate [their] discussion of how Clustervision could potentially be used to arrive at more satisfactory clusterings”. The “Bob Ross Paintings” dataset hereinafter referred to as the paintings dataset is a dataset about the 403 paintings produced on the PBS show “The Joy of Painting”. It includes 67 binary values per painting specifying the existence of features such as trees, water, mountains, and weather elements, as well as meta information about each painting such as number of used colors and the TV episode name.

#### 5.1.1 Preparations

For this analysis workflow, we developed and added the following R-apps to the VisR framework. It is worth noting that by taking advantage of the available functionality in R for analyses such as clustering and dimensionality reduction, and using VisR’s app development API, developing these R-apps was done in a matter of hours.

**FIGURE 5:**
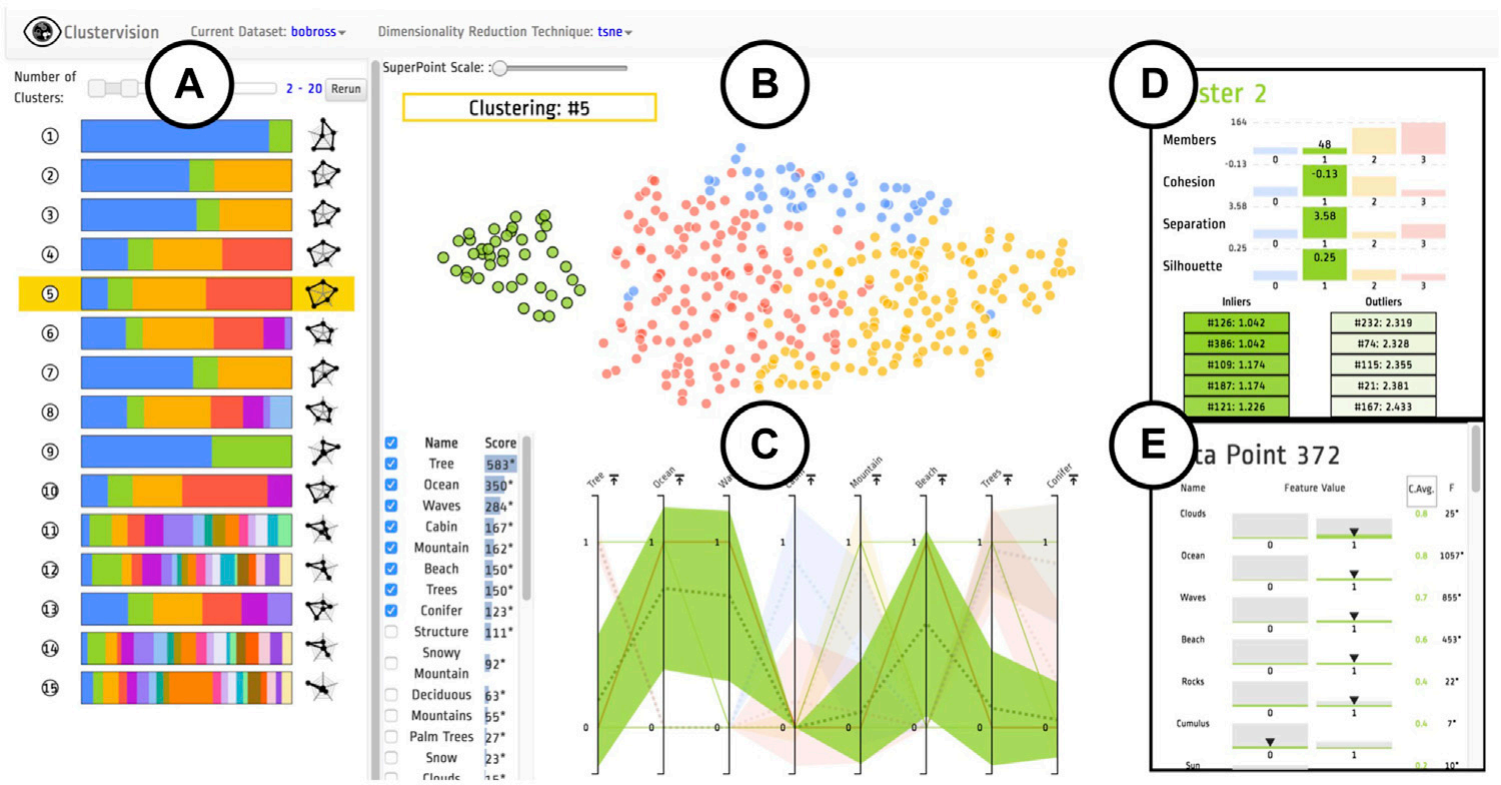
An overview of the Clustervision on “The Joy of Painting” dataset [source: Kwon et al. (2018) Figure 1]. **(A)** *Ranked List of Clustering Results* sorted by the aggregated quality measures; **(B)** *Projection* shows a selected clustering result colored according to corresponding clusters; **(C)** *Parallel Trends* show the trends of feature values of data points within corresponding clusters; **(D)** *Cluster Detail* shows quality measures of a selected individual cluster; **(E)** *Data Point* shows the feature value distribution of the selected cluster.

• The Clustering app, implements four clustering methods: k-means, spectral clustering Karatzoglou et al. (2004), hierarchical clustering and DBSCAN Hahsler and Piekenbrock (2018). A user can select the input data columns to which the clustering should be applied. The app will output two data tables: A table of cluster IDs which has one column and as many rows as the input data table, and a data table of clustering quality metrics computed using the NbClust R package Charrad et al. (2014). It also plots a static scatter plot and bounding hull of the dimensionality reduced tSNE projection of the input columns, colored by the cluster IDs. Examples of such output plots are shown in Figure 4E.
• The DimRed app, includes dimensionality reduction methods such as PCA, MDS, and tSNE. It allows users to select several numerical columns on a data table and computes a 2D projection using the specified method.
• The RadarChart app, plots a Radar Chart of user specified numerical columns, an example of which is shown in Figure 4G. This was made to replicate the visual encoding of clustering quality metrics in ClusterVision as shown in Figure 5A.

Prior to starting the parameter space analysis, we imported the paintings dataset in VisR and used the DimRed R-app to compute two dimensional projections of the 67 features. We ran the R-app using the three available dimensionality reduction methods, resulting in 6 new columns to be added to our original input table (two dimensions for each method). This was done in order to better visualize the clustering results in the exploration step.

#### 5.1.2 Parameter sampling

To perform parameter sampling on the Clustering app, we first drag-and-dropped it into the VisR workspace and drag-and-dropped the paintings dataset into its view. We then switched the parameter view to ModEx view mode. Figure 4A shows the ModEx view mode and the UI controls to specify the desired parameter ranges for each clustering method. To be consistent with the Clustervision case study, we selected the three clustering methods: k-means, hierarchical, and spectral clustering. For all methods we set the range of the “k” parameter (number of clusters) to [2, 19] and enabled all options for their categorical parameters “algorithm”, “method”, and “kernel”. We finally specified the directory to store the results, set the number of runs to 1,000 and started the parameter sampling process. Running clustering for 1,000 random parameter combinations took about 2 h on a 2012 Macbook Pro. Once done, the output directory will contain the following:

• The paramsInfo table with the names and types of the parameters.
• The runsInfo table with one row for each run (1,000 rows total) and one column per parameter specifying the value assigned to the parameter for each run.
• Two data tables (cluster_ids and quality_criteria) per run (total of 2 × 1,000 data tables)
• One output image per run (total of 1,000 images).

#### 5.1.3 Derivation

The next step after parameter sampling was computing the derived outputs. We added the ModEx app to the workspace and specified the directory of runs to the output directory created in the previous step. For this case study, we ran two passes of the derivation step: In the first pass we chose the derivation method “Number of class labels” on cluster_ids table. This added a new column Num(cluster_ids) to the runsInfo table, specifying the number of clusters generated for each run. Note that even though the number of clusters was specified as an input parameter, it was possible for some clustering methods to converge to fewer number of clusters. In the second pass, we chose the derivation method “Take first row” and specified the quality_criteria table as shown in Figure 4B. We picked the five clustering quality metrics used in the Clustervision including Calinski-Harabaz index, Silhouette Coefficient, Davies-Bouldin index, Gap Statistic and SDbw as well as the mean normalized value as an aggregated quality metric. This added one column per quality metric to the runsInfo table.

#### 5.1.4 Exploration

In this section we will compare the exploratory analysis of the paintings dataset using ModEx and Clustervision. The main purpose of this case study is to demonstrate how custom parameter space analysis workflows such as Clustervision can be prototyped and developed in our general purpose framework. So we will not be focusing on the effectiveness of Clustervision’s proposed workflow and rather will discuss how much of it could be recreated and possibly enhanced in our framework.

The ranked list view of Clustervision (Figure 5A) shows different clustering results. A set of horizontal colored stripes show a representative of a clustering where the length of the colored stripes represents the number of data points (e.g., paintings) in a cluster. The User can adjust a range slider to focus on clustering sizes relevant to their analysis. On the right of each bar, a radar chart is shown consisting of a sequence of five spokes, each representing one of the quality metrics. The length of each spoke from the center is proportional to the normalized score of the quality metric and they are connected to form a polygon shape.

The initial configuration of the exploration interface of ModEx after start up, contains the views in Figures 4C–F. The Data view in Figure 4C is populated with three data tables. The input table is the input data set, i.e., the meta data of the paintings. The runsInfo table is the parameter values as well as the derived outputs for each run. The Run Output table contains the results for the currently selected run (i.e., cluster IDs) concatenated to the input table.

**FIGURE 6:**
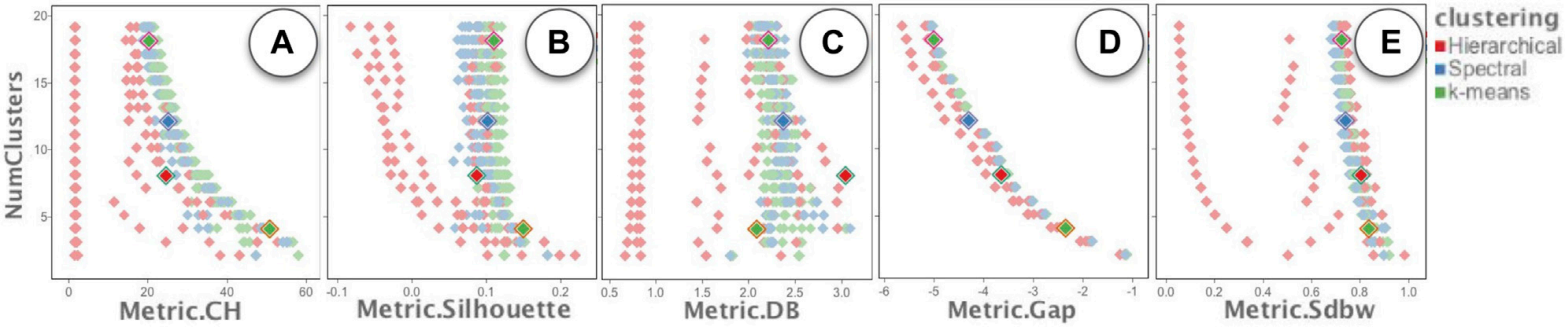
Exploring the quality of the clusterings using different clustering quality metrics: **(A)** Calinski-Harabaz index, **(B)** Silhouette Coefficient, **(C)** Davies-Bouldin index, **(D)** Gap Statistic, and **(E)** SDbw.

The “Runs Overview” in Figure 4D takes the runsInfo table as input. We were not able to create the exact user interface of the ranked list view of the Clustervision in ModEx without creating a new app. However to reproduce a similar functionality, we set the x-axis of the “Runs Overview” to the mean normalized metric and the y-axis to the number of clusters. A quick browsing revealed that the clusterings with the higher quality metrics are those with lower number of clusters. This was consistent with the findings in the Clustervision. Alternatively a user could also set the x-axis to any of the metrics to study the clusters based on that metric. This is shown in Figure 6. A user could click to select the run(s) with the desired quality metric and number of clusters. The image output of the selected runs would show in the “Output of Selected Run(s)” (Figure 4E). Also the Run Output data table will update to the output of the main selected run.

Users can also use the sliders in the “Runs Distribution” view (Figure 4F) to filter the runs to a smaller group, for example, to focus on specific range of cluster numbers or specific clustering methods. We will further demonstrate the use of this feature in the next section.

To reproduce the radar charts in the ranked list of Clustervision, we drag-and-dropped the RadarChart R-app to our workspace and assigned the runsInfo table as its input as shown in (Figure 4G). Within the RadarChart app’s parameter view, we selected the five columns of quality metrics as the dimensions to be shown on the plot and enabled the “Auto Run” option of the R-app. Every time one or more runs were selected in the “Runs Overview” the RadarChart would refresh to show the polygons formed by the quality metrics of the selected run(s).

The Projection view of Clustervision shows input data points (here the paintings) in a two dimensional tSNE projection, resembling a scatter plot, as shown in Figure 5B. When users select a clustering result from the Ranked List of Clustering Results view (Figure 5A), the data points in the Projection view are colored to match its cluster.

To recreate this behaviour in ModEx, we drag-and-dropped the Scatter Plot app of the VisR framework to the workspace and assigned the “Runs Output” table as its input. As shown in Figure 4H, we then assigned the cluster ID column as the point colors and the tSNE1 and tSNE2 columns to the *x* and *y*-axis (we could also use the MDS or PCA columns). Every time the user was selecting a run in the “Runs Overview”, the Run Output table would be updated and as a result the scatter plot would refresh to draw the points colored by the new cluster IDs for the selected run. In addition we also found it useful to set the image column to the tooltip of the scatter plot so it would show a thumbnail of the paintings as the user moved over the data points.

The Parallel Trends view of Clustervision shown in Figure 5C uses vertical axes to represent each feature of the data points. It draws a line per cluster to show the mean values for each cluster for the corresponding data feature and an area path to represent standard deviation or 95% confidence intervals. We used the existing Pararallel Coordinates app of the VisR framework to recreate a similar visual encoding as shown in Figure 4I. Similar to the tSNE scatter plot, we assigned the Run Output table to the input of the Parallel Coordinates app. We then set it up to only show the 67 columns corresponding to the painting features and to use the cluster ID column for coloring the lines. The Parallel Coordinates app, offers an aggregation mode which allows drawing aggregate values such as the mean, median, standard deviation and quartiles for each group of data based on the values in the column specified for the color. We used this mode to show the mean values of the data features for each cluster and specified the column sorting through clicking on the legend (clusterID).

The Clustervision has the option to rank input features in order of importance based on the ANOVA F-Value. The existing Parallel Coordinates app in the VisR framework did not include this feature. Although it was technically possible to develop this new feature in the app, we did not invest on doing this. However, just by sorting features based on the aggregated value of each cluster, we could already detect similar patterns reported by Clustervision.

**FIGURE 7:**
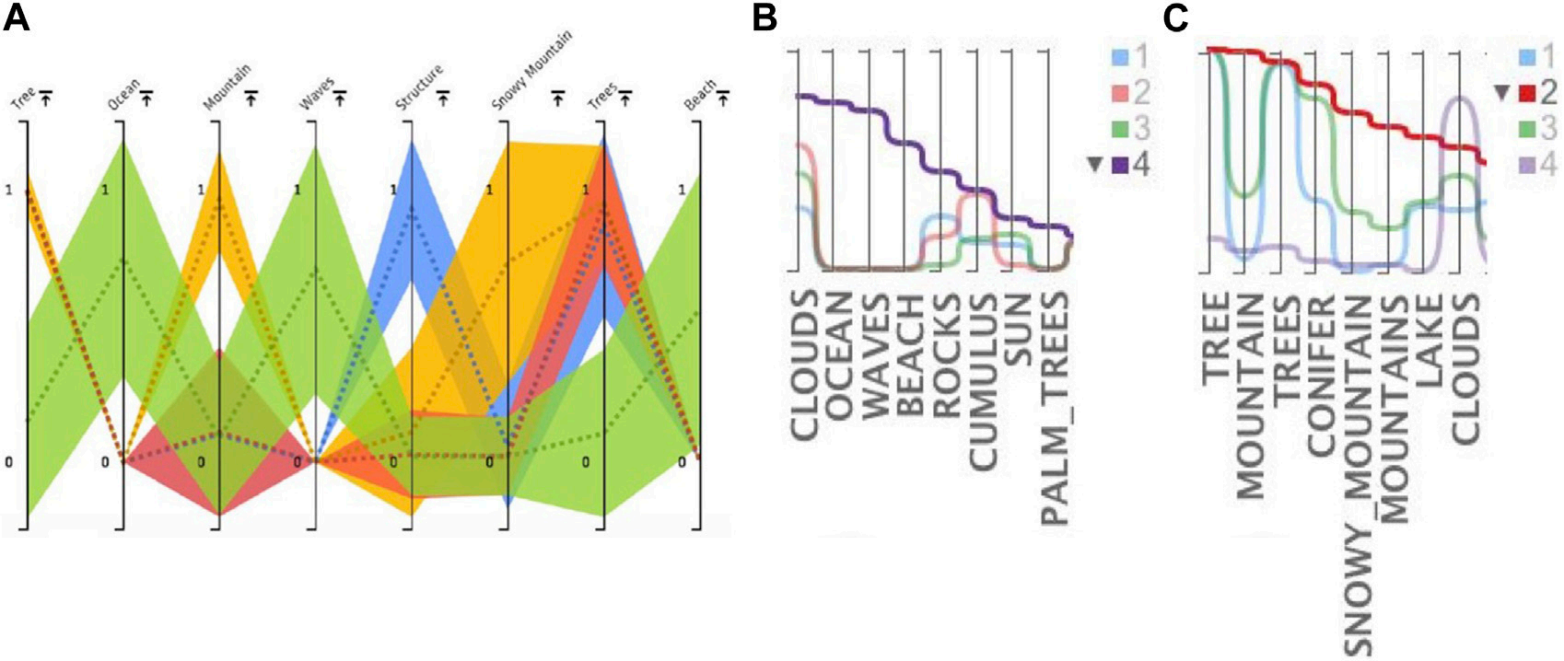
Exploring top features of a selected clustering in Clustervision and ModEx: **(A)** Top features in Clustervision based on the ANOVA F-Value; **(B, C)** Top features in ModEx based on sorting by aggregated value for cluster 4 (purple) and cluster 2 (red).

Figure 7A, shows the top 8 most important features in Clustervision. Figures 7B, C show the top 8 features sorted by the aggregated value of cluster 4 (purple) and cluster 2 (red) respectively. We can see that the top features of the purple cluster (ID: 4) of Figure 7B include “Ocean”, “Waves” and “Beach” which are also contained in the green cluster of Figure 7A. Also the red cluster (ID: 2) of Figure 7C includes “Mountain” and “Snowy Mountain” features which are also contained in the yellow cluster of Figure 7A. Additionally we see that “Tree” and “Trees” features are prominent in all but one of the clusters, the green cluster of Figure 7A and the purple cluster of Figure 7C.

The two remaining views of Clustervision “Cluster Detail” (Figure 5D) and “Data Point” (Figure 5E) were not added to our workspace as we did not have existing apps in VisR that could reproduce them. Although it was not technically difficult, we did not create two new R-apps for this purpose as they would have been apps only specific to this case study and we believed they would not contribute in further demonstrating the flexibility and generality of our tool.

#### 5.1.5 Further analysis

**FIGURE 8:**
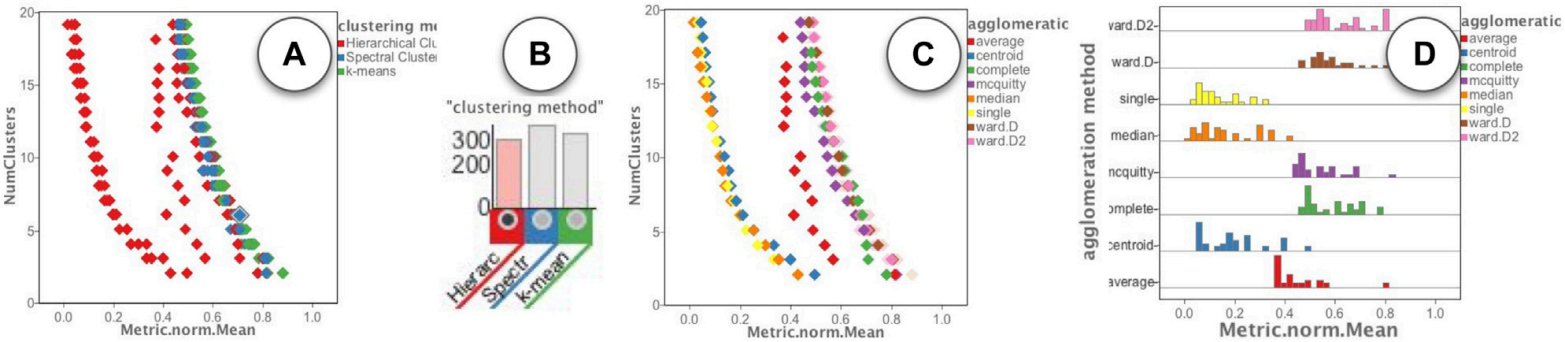
Visual analysis of the clusterings: **(A)** Observing the group of runs with low quality are from hierarchical clustering method; **(B)** filtering selecting runs using the hierarchical clustering method; **(C,D)** Observing the group of runs with low quality use “single”, “median” and “centroid” values for the “agglomeration method” parameter.

While exploring the runs in the “Runs overview” of Figure 4D, we noticed a fairly large group of runs (in red color) that had a much lower value of the mean quality metric regardless of the number of clusters. To further analyze these runs, we used the filters in the “Runs Distribution” view of Figure 4F. The steps of our analysis are shown in Figure 8. First, as shown in Figure 8A, we noticed that all the low quality clusters were using the “Hierarchical” clustering method (red color). So we used the corresponding histogram filter to select those runs, as shown in Figure 8B. We then browsed the other parameters for the remaining runs and noticed a distinct pattern for the “agglomeration method”. As shown in Figures 8C, D the values of “single”, “median”, and “centroid” (yellow, orange, and blue colors), were used in the low quality runs. These runs could then easily be removed by filtering out those values.

### 5.2 Case study: differential gene expression

In this section we demonstrate the effectiveness of ModEx using a case study performed in collaboration with a group of biologists.

#### 5.2.1 Related work in bioinformatics

Our initial motivation for developing ModEx came after our collaborations with biologists to develop methods for understanding biological data. Recent advances in next-generation sequencing technology have significantly progressed understanding of genomics and molecular biology. Most of the related bioinformatics research has focused on statistical and computational methods for processing and analysis of the biological data. Some of the most used ones are limma Smyth (2005), edgeR Robinson et al. (2010), and DESeq Anders and Huber (2010); Love et al. (2014) which will be further discussed in our case study in Section 6.

There have also been several works done to provide guidance in selecting the suitable algorithms and parameters for those computational methods based on the dataset and the goals of the analysis. Among them, a group that are most related to this work are approaches that combine cluster analysis with interactive visualization techniques to facilitate analysis and understanding of large data. Examples of such methods which provide visual interfaces for tasks such as comparison of several clustering results are Genesis Sturn et al. (2002), HCE Seo and Shneiderman (2002), Mayday Battke et al. (2010), XCluSim L’Yi et al. (2015), MLCut Vogogias et al. (2016), VisExpress Simon et al. (2017) and Kern et al.Kern et al. (2017).

#### 5.2.2 Introduction

Differential gene expression (DGE or DE) analysis is one of the common analysis in genome biology and is used to identify genes (or other genomic features) that are expressed in significantly different quantities in distinct groups of samples Huss (2014). These data are acquired using genome sequencing technologies (e.g., RNA-seq) from samples collected in different biological conditions such as drug-treated vs. controls, diseased vs. healthy, different tissues or different stages of development. Bioinformaticians have developed several computational methods such as Limma Smyth (2005), edgeR Robinson et al. (2010), DESeq Anders and Huber (2010) and DESeq2 Love et al. (2014) that employ various statistical models to identify differentially expressed (DE) genes. These methods take two groups of gene expression datasets (with two or more replicates per group) as input and assign a label of either “0”, “-1”, or “+1” to each gene. A none DE gene is labelled “0”, while a DE gene is labelled “+1” when it is detected to be expressed in significantly higher quantity in the second group (a.k.a. upregulated) or “-1” when it is the other way around (a.k.a. downregulated).

**FIGURE 9:**
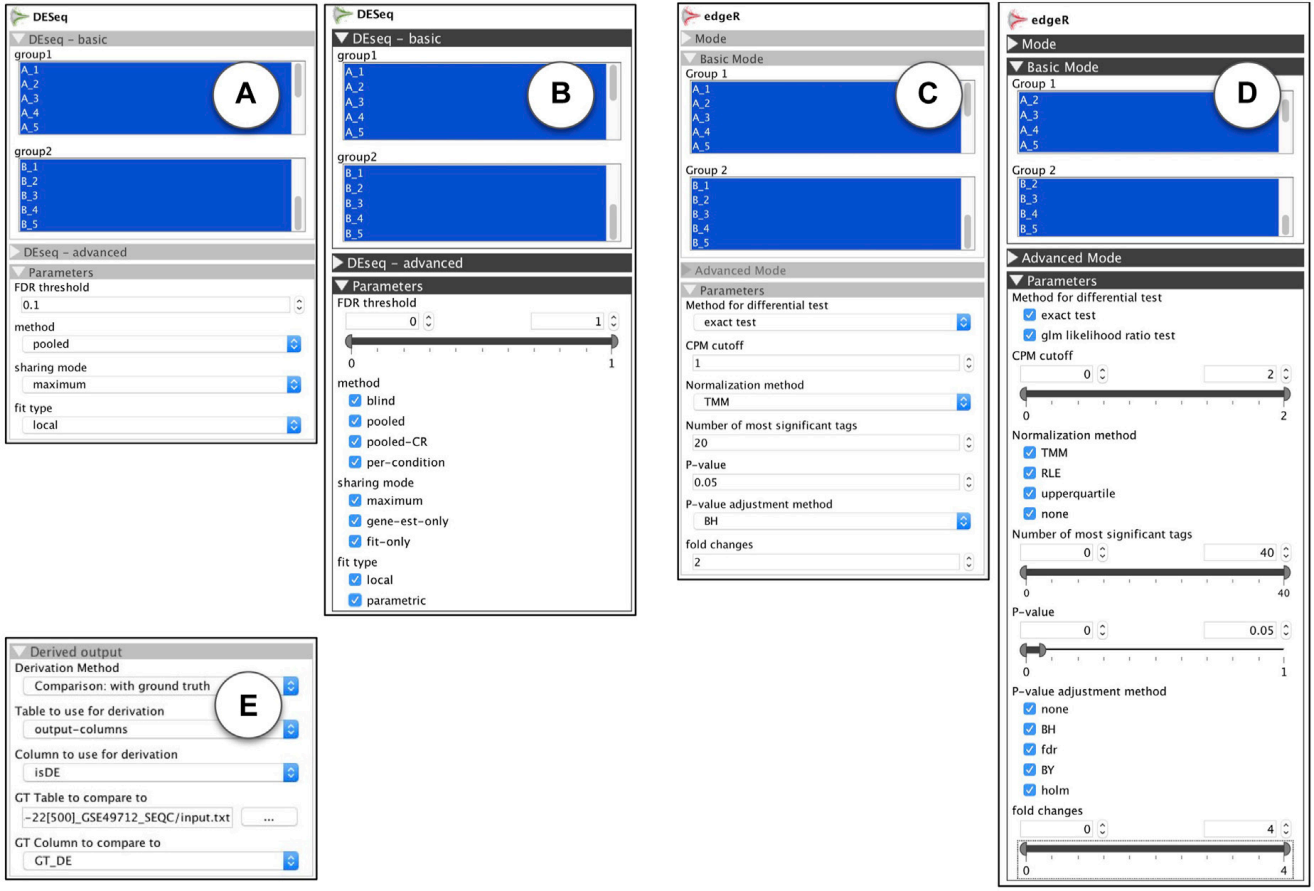
R-apps for differential expression analysis: **(A)** DESeq: standard view **(B)** DESeq: ModEx view **(C)** edgeR: standard view **(D)** edgeR: ModEx view **(E)** Derivation method “Comparison: with ground truth”.

Our users were interested in using DESeq Anders and Huber (2010) and edgeR Robinson et al. (2010) as the two most commonly used methods for DE analysis Anders et al. (2013). R-apps for these methods were already incorporated in VisR as shown in Figures 9A, C. One of the main questions that biologists had when using these apps, was whether the genes were classified as DE through a stringent computer model (i.e., specificity) or more tolerant one (i.e., sensitivity). They also wanted to have control over the sensitivity. That was because sometimes in earlier stages of their studies they wanted to cast a wide net to identify a large set of possible candidates and later perform more rigorous focused analysis (e.g., pathway analysis or wet lab analysis) to narrow down the results, while other times they wanted to be most confident about the detected DE genes.

#### 5.2.3 Parameter sampling

In order to study the effect of parameters on the sensitivity of DE analysis methods, we used a benchmark gene expression dataset called SEQC (a.k.a. GSE49712) Rapaport et al. (2013). The data was prepared under two biological conditions, and for each condition, the sample collection and measurement was repeated 5 times (biological replicates). In addition to the genetic material from tissues, a mixture of 92 synthetic genes, called spike-in, was added to each sample with known abundance, so the DE states of those genes were known in advance. For 25% (23) of the spike-in genes the ratio of genetic material added to both groups of samples was identical, so they were expected to be detected as non-differentially expressed. For the remaining 75% (69) spike-in genes the ratio was different (0.5, 0.67 or 2) so they were expected to be detected as differentially expressed. The final sequenced and processes dataset was stored in a data table with 21,716 rows (one per gene, 92 of which were spike-in), 10 columns of gene expression values (5 for each of two biological conditions) and one column “GT_DE”, indicating the ground truth DE state, with a value of *TRUE* or *FALSE* for the 92 spike-in genes, and *NA* for the remaining of the genes. First we used the ModEx view for each app shown in Figures 9B, D and performed the DE analysis for 500 runs with random combinations of input parameters. The output of each run was the computationally determined DE state for each gene. This was stored as a data table with one row per gene, and two columns, “DE” with a value in {-1, 0, +1} and “isDE” with a value of *FALSE* when DE = 0 or *TRUE* when DE = −1 or +1.

#### 5.2.4 Derivation

Our goal was to compare the actual DE state of the control spike-in genes, with the DE state computed by the DESeq and edgeR apps given a set of parameters. We studied each of these apps separately. For derivation, we used the “Comparison: with ground truth” derivation method as shown in Figure 9E and set it to use the “isDE” column of the app’s outputs with the “GT_DE” column of the input dataset. Each gene’s computed DE condition,“isDE”, could take one of the two values of *TRUE* or *FALSE* and the ground truth DE condition, “GT_DE”, could take one of the three values of *TRUE*, *FALSE*, or *NA*, so there were a total of six combinations possible for the output of each run for each gene:

• isDE = TRUE & GT_DE = TRUE: indicating a true positive
• isDE = TRUE & GT_DE = FALSE: indicating a false positive (type I error)
• isDE = FALSE & GT_DE = FALSE: indicating a true negative
• isDE = FALSE & GT_DE = TRUE: indicating a false negative (type II error)
• isDE = TRUE & GT_DE = NA: possibly a DE gene
• isDE = FALSE & GT_DE = NA: possibly a non-DE gene

The derivation step counted the combinations for each run. This resulted in 6 columns that were added to the runsInfo table. Each column contained the count of genes for one of the 6 combinations. For example, the column “n(isDE = TRUE & GT_DE = TRUE)” contained the number of true positives for each input parameter combination.

#### 5.2.5 Exploration

We will now discuss our findings using the ModEx exploration interface, first for the DESeq app and then for the edgeR app. For studying the sensitivity of a classifier model, the “receiver operating characteristic” plot (ROC plot) is one of the most well known visualizations. The ROC plot is created by plotting the true positive rate (TPR) against the false positive rate (FPR) at various parameter settings Wikipedia contributors (2018).

To get a ROC plot, we selected the “n(isDE = TRUE & GT_DE = FALSE)” (to represent the FPR) as the x-axis and the “n(isDE = TRUE & GT_DE = TRUE)” (to represent the TPR) as the y-axis of the plots. Note that these represented counts (not rates) as they were computed by the derivation method without the knowledge of the total counts. To compensate for this, we set the x-axis maximum to 23 (the number of non-DE spike-in genes) and the y-axis maximum to 69 (the number of DE spike-in genes).

**FIGURE 10:**
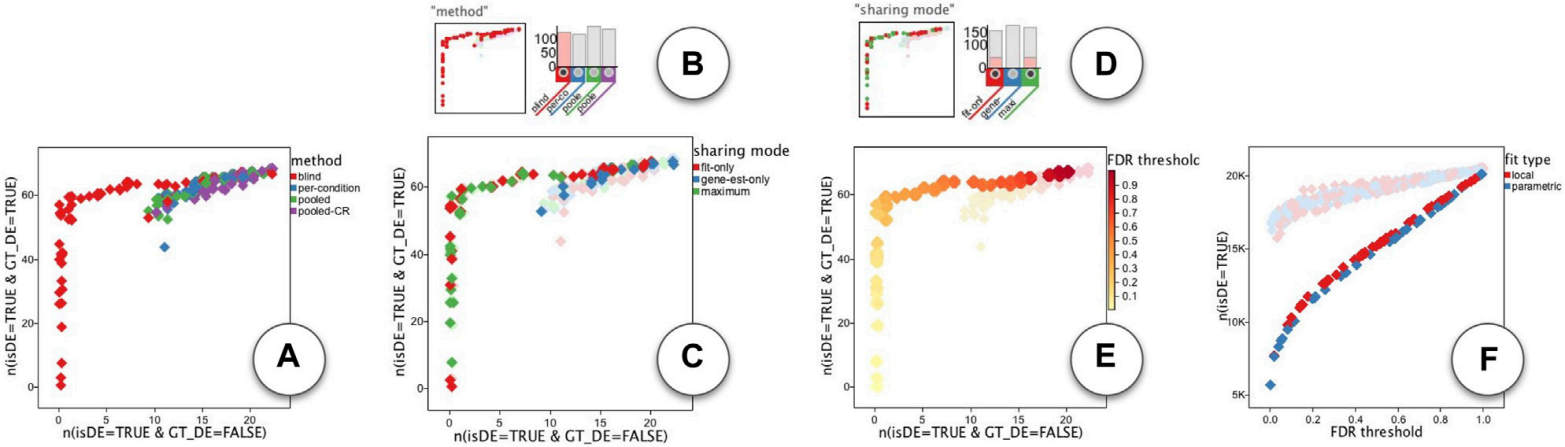
ModEx exploration of DESeq app: **(A)** ROC plot of runs colored by the “method” parameter. **(B)** selecting runs that use the “blind” value for the “method” parameter. **(C)** ROC plot of selected runs colored by the “sharing mode” parameter. **(D)** selecting runs that use the “fit-only” or “maximum” value for the “sharing mode” parameter. **(E)** ROC plot of filtered runs colored by “FDR threshold” parameter. **(F)** Correlation of number of detected DE genes with the “FDR threshold”.

Figure 10A shows one of the ROC plots for DESeq runs. Each point represents one run (i.e., an input parameter combination) and the color represents the value used for the categorical parameter “method”. In a ROC analysis, the optimum points are those for which there is no other point with both lower FPR and higher TPR. These points form a set known as the *Pareto set* and are referred to as the *Pareto Frontier* in a ROC plot. Browsing through the ROC plots, we observed that a large group of points in the Pareto set were colored red as shown in Figure 10A. Those were the runs that used the value “blind” for the “method” parameter, so we used the histogram filter Figure 10B to select them. We then observed that the categorical parameter “sharing mode” was determining the Pareto set, more specifically the two values of “fit-only”(red) and “maximum”(green), as shown in Figure 10C. So we used the histogram filter once more Figure 10D to select runs using those two values. The remaining points were those either in the Pareto set or very close to it. For these points, the parameter “FDR-threshold” had a clear correlation as shown in Figure 10E. Runs with a lower value for “FDR threshold” had a low FPR and TPR, while those with a higher value for “FDR threshold” had also high FPR and TPR. A value of near 0.4 seemed to be a reasonable value to have keep the FPR low at 4% $\left(\frac{1}{23}\right)$ and TPR high at 87% $\left(\frac{60}{69}\right)$ . Last but not least, a forth parameter “fit type” seemed to have a slight effect on the number of detected DE genes. As shown in Figure 10F, when plotting the total number of DE genes vs. the FDR threshod, we could see that a value of “local” resulted in detecting slightly more DE genes compared to a value of “parametric”.

**FIGURE 11:**
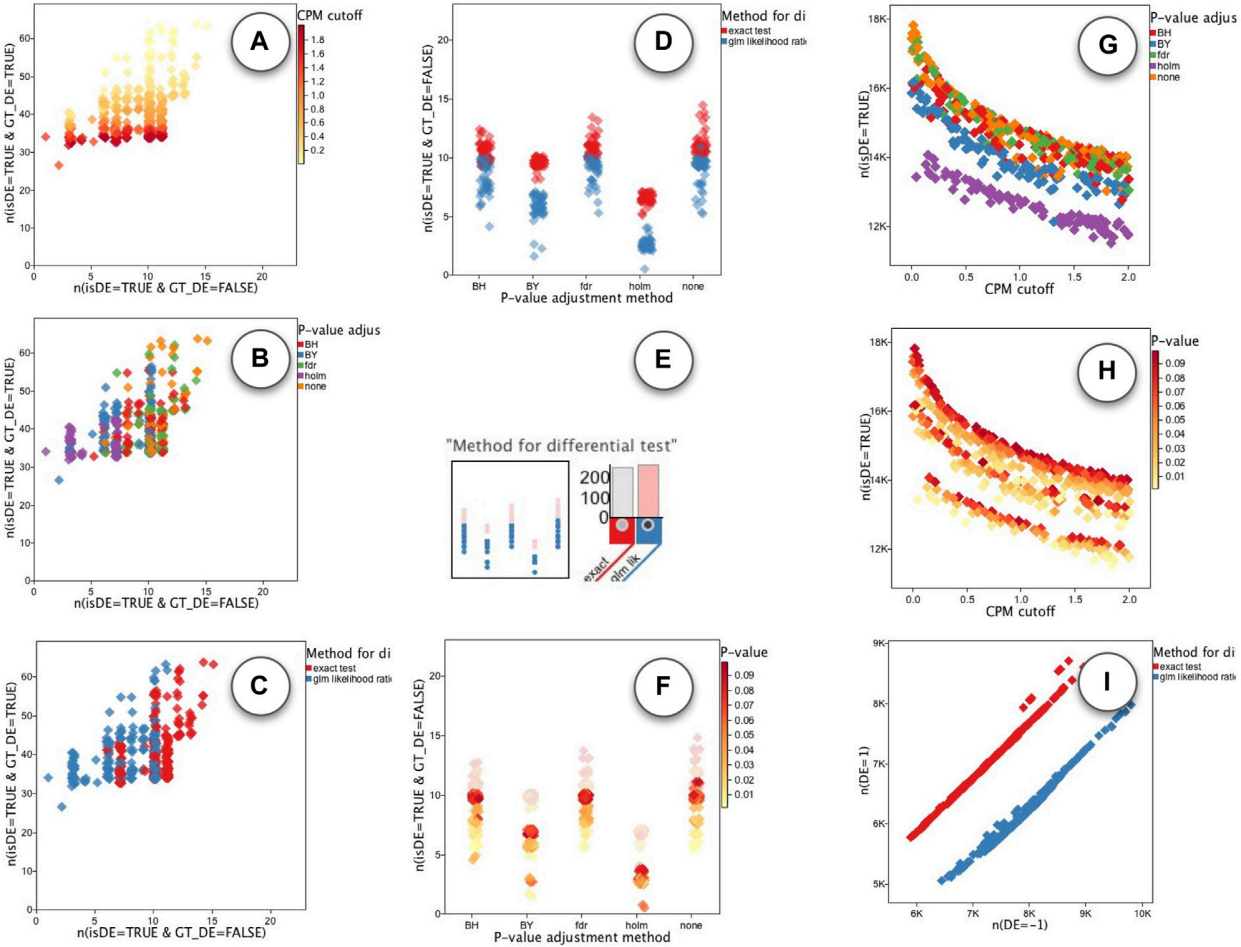
ModEx exploration of edgeR app. ROC plot of runs with the x-axis set to FPR and the y-axis set to TPR and colored by the parameters: **(A)** the “CPM cutoff”, **(B)** “P-value adjustment method”, and **(C)** “Method for differential test”. **(D)** effect of “P-value adjustment method” (x-coord) and “Method for differential-test” (color) parameters on the FPR (y-coord). **(E)** Selecting runs using “glm likelihood ratio test” value. **(F)** Positive correlation between “P-value” (color) parameter and FPR (y-coord). **(G)** Effect of “CPM cutoff” (x-coord) and “P-value adjustment method” (color) on the number of detected DE genes (y-coord). **(H)** Effect of “CPM cutoff” (x-coord) and “P-value” (color) on the number of detected DE genes (y-coord). **(I)** “Method for differential test” parameter biases the detection of downregulated (x: n (DE = −1)) and upregulated (y: n (DE = 1)) genes.

We performed a similar ROC analysis on the runs of the edgeR app. Unlike DESeq, we initially did not see a single parameter that would clearly define the Pareto set. As shown in Figure 11A The “CPM cutoff” parameter had a negative correlation with the TPR, but did not directly effect the FPR. On the other hand, the “P-value adjust method” parameter had some effect on the FPR but not directly on TPR: As shown in Figure 11B the value “holm” (purple) resulted in runs with lower FPR. The “Method for differential test” parameter was another parameter that had some effect on the FPR: As shown in Figure 11C, runs with “glm likelihood ratio test” parameter value (blue) resulted in relatively lower FPR than the runs with “exact test” value (red). To further explore the parameters affecting FPR, we set the plots to show FPR vs. “P-value adjustment method”. The plot that used “Method for differential test” parameter as the point color, had a visible association. As shown in Figure 11D the “holm” value for “P-value adjustment method” parameter (x-coordinate) resulted in overall reduced FPR among other groups, but within each group, the runs using “glm likelihood ratio test” parameter value (blue) had always less FPR than those using the “exact test” parameter value (red). Note that there is a slight jitter on point coordinates to handle over-plotting. So we used the histogram filters (Figure 11E) to select the runs with “glm likelihood ratio test”. This made it easy to observe that the “P-value” method had a positive correlation with FPR as shown in Figure 11F.

In order to explore the effect of parameters on the total number of detected DE genes, we set the plots to show the number of detected DE genes, “n(isDE = TRUE)”, vs. the “CPM cutoff” parameter. As shown in Figures 11G, H, the number of detected DE genes decreases by increasing the value of “CPM cutoff”. The “holm” value for “P-value adjustment method” parameter detected the least number of DE genes (Figure 11G). For each selected value of the “P-value adjustment method” parameter the “P-value” parameter had a slight positive correlation with the number of detected DE genes (Figure 11I).

We also noticed that the “Method for differential test” parameter introduced a bias in detecting upregulated (DE = 1) vs. downregulated (DE = −1) genes. As shown in (Figure 11H), when using “exact test” value (red), more upregulated (DE = 1) genes were detected. In contrast, using the “glm likelihood ratio test” (blue) showed a bias toward detecting more downregulated (DE = −1) genes.

To summarize, we find that the DE results of the DESeq method seem to be closer to an ideal ROC, than the edgeR method. For DESeq it seems the parameter combination of {‘method’: ‘blind’, ‘sharing mode’: ‘fit-only’} should be used and the ‘FDR threshold’ can be used to control the specificity of the detection method.

### 5.3 Case study: neural network playground

Neural networks have gained an unprecedented popularity in recent years thanks to their effectiveness in many application domains. In the context of machine learning, the term “parameter” is referred to model variables (such as weights and biases) values of which are learned automatically through a training process. For the constant values that are specified manually to fine tune a model the term “hyperparameters” is used. To solve problems with neural-networks, a machine learning expert would choose a network architecture and set the hyperparameters. These choices are based on heuristics and the expert’s knowledge of the specific problem, and often involve a trial-and-error strategy.

The Neural Network Playground Smilkov et al. (2017) (hereinafter referred to as the Playground) is a web-based interactive application that captures the essence of this task by allowing users to create simple neural networks, visualize the learning progress, and modify hyperparameters. In this section we demonstrate the ModEx workflow and system features using a problem inspired by the Playground application.

**FIGURE 12:**
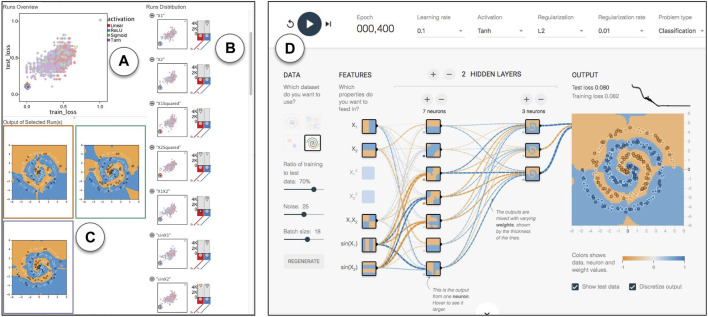
Linked views in ModEx parameter space exploration: **(A)** “Runs Overview” showing a scatter plot of the runs for the selected derivations, (i.e., “train_loss” and “test_loss”) and colored by the currently selected parameter (i.e., “activation”). **(B)** “Runs Distributions”, showing a list of histogram distributions for input parameters and derived output together with a scatter plot colored by the corresponding parameter. **(C)** Image output of the runs selected in the “Runs Overview” plot. **(D)** Neural Network Playground launched in a browser and initialized automatically after clicking on a run.

Figure 12D shows an example view of the Playground application. The Playground offers two problem types: A binary classification problem and a regression problem. Here we will be focusing on the binary classification problem as it is the more complex one. To generate the dataset for the classification problem, a user selects from one of 4 dataset shapes (Gaussian, Circle, Xor, and Spiral) and a noise value between 0 and 50 and the Playground generates a random two dimensional dataset. The resulting dataset has 200 points which are divided equally into two class labels, −1 and +1, visualized with orange and blue respectively.

To build a neural network, a user can add between 0 and 6 hidden layers (excluding the input and output layers) and set the number of neurons in each layer between 1 and 8 and a fully connected network architecture will be constructed. For the input layer, a user can independently toggle any of the seven input features *X*_1_, *X*_2_, $X_{1}^{2}$ , $X_{2}^{2}$ , *X*_1_ *X*_2_, sin(*X*_1_), and sin(*X*_2_). Before training, several hyperparameters can be specified: “Ratio of training to test data”, “Batch size”, “Learning rate”, “Activation” function, “Regularization” method, and “Regularization rate”. Once done, a user can start the training and the Playground will visualize the results in real time after each training epoch.

#### 5.3.1 Parameter sampling

We were interested in using ModEx to explore the parameter space for this application. The Playground is developed in TypeScript using D3 so creating a VisR app directly from the code was not practical. Instead we decided to reimplement the playgrounds machine learning model using Python and TensorFlow Abadi et al. (2016) to be able to run the tool in batch mode with different combinations of parameters. At the time of the implementation, we were not aware of the existence of a recent R implementation of TensorFlow so the parameter space sampling is not done in ModEx, but the results were stored in the same structure described in Section 4.2. We made an effort to make our implementation as close as possible to the online demo, however, there were occasionally some subtle differences between the outcomes of the two which we believe were the result of the differences between the implementations of the simpler online version of the neural network library compared to the standard Python version as well as the differences in the random number generators. As input, we created one of each of the four dataset types with a noise level of 25. Each input dataset has 200 data points, each with values for the seven input features in the Playground application, as well as its “true” label. For each dataset, we ran the network with 2,500 random combinations of parameters and recorded the results at 4 epochs: 50, 100, 200 and 400. So at the end, for each input dataset, we had a total of 10,000 records in the runsInfo table. For each run we saved an output table of the predicted labels as well as a 2D scatter plot image of the input data points and the predictions, examples of which are shown in Figure 12C. The points are coloured based on their true label (orange for −1 and blue for +1) and their inclusion in the training or the test set is indicated with a white or black stroke color respectively. The background color of the plot shows what the network has predicted for a particular area after each run.

#### 5.3.2 Derivation

The neural network playground script collects several measures after running each configuration of the network for the specified number of epochs. Those include “total time”(training), “mean time”, “train loss”, “test loss”, “train TPR” (True Positive Rate), “train FPR” (False Positive Rate), “test TPR”, and “test FPR”. We could derive some of these measures (e.g., TPR and FPR) within ModEx, but others could only be collected during the training of the network. As such, this example demonstrates how ModEx can still be utilized for parameter exploration of applications developed outside the VisR framework.

#### 5.3.3 Exploration

We will now discuss some of the insights from the parameter space exploration of the neural network outputs. We will specifically focus on addressing the feedback from the users of the Playground application stated by Ha Ha (2016): “People started experimenting with different neural network configurations, such as how many neural network layers are actually needed to fit a certain data set, or what initial features should be used for another data set. Which activation functions work better for which dataset?”.

We started by exploring the scatter plots of train loss vs. test loss (Figure 12A), with runs colored by different hyperparameters (Figure 12B). We selected runs that best fit each dataset (i.e., runs that led to low train loss and test loss) by clicking on the points on the bottom left of the scatter plot (Figure 12C). The values of the hyperparameters for the selected runs would also be indicated by a pointing down triangle above the histogram for each hyperparameter. We could also link the workflow to a simple custom R-app that launches the Playground with the hyperparameter configuration of the selected run in the URL (Figure 12D).

**FIGURE 13:**
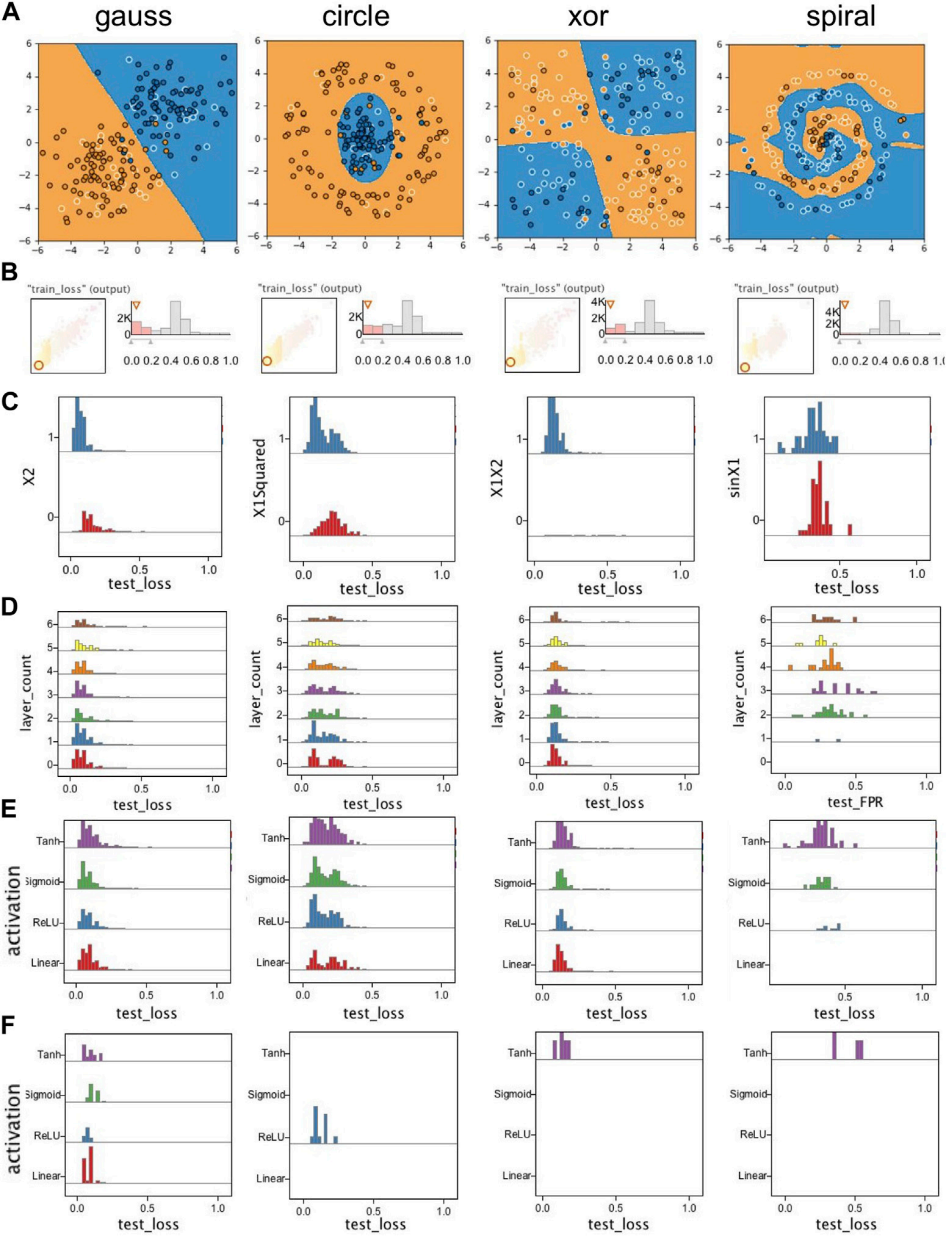
Selected plots from exploration of runs for the Playground case study: **(A)** Example outputs from converged runs for each dataset; **(B)** Filtering out runs with high train_loss; **(C)** Example of input features showing high correlation with converged (low loss) runs; **(D)** Exploring effect of the number of hidden layers; **(E)** Exploring the effect of activation functions; **(F)** Exploring the effect of activation functions on runs that only used *X*_1_ and *X*_2_ as input features. Except for the gauss data, a nonlinear activation function is needed to train a successful classifier.

Even though this workflow helped us get some initial intuitions, it was still difficult to get any global insights as we did not see any obvious patterns in most of the scatter plots. This was partially due to over plotting but also since the effectiveness of the runs was affected by a combination of a large number of hyperparameters rather than a few individual ones. We were interested in studying the hyperparameters for the runs which converged and correctly fit the classes in each data (i.e., runs with small loss). Example outputs of such runs are shown in Figure 13A. So we used the filters shown in Figure 13B to filter out the runs with a high training loss (>0.2) . The percentage of the remaining effective runs were highest for the “gauss” dataset (24%) and lowest for the hardest “spiral” dataset (1%) while the “circle” and “xor” both had around 19% runs remaining after filtering. This was not surprising given the relative difficulty of the classification for each dataset. Next we set the x-axis to test_loss and browsed through different hyperparameters for the y-axis. Looking through the input features we could see distinctive patterns for each dataset. The input features that had the most effect on converged runs were *X*_1_ and *X*_2_ for the “gauss”, $X_{1}^{2}$ and $X_{2}^{2}$ for “circle”, *X*_1_ *X*_2_ for the “xor” and sin(*X*_1_) and sin(*X*_2_) for the “spiral” datasets. Examples of each case are shown in Figure 13C.

We then looked at the effect of the number of layers. As shown in Figure 13D, for the “spiral” dataset a minimum of two layers were required, however the runs for other datasets could converge even without any hidden layers. We could even notice that there were slightly more converged runs when the number of layers were low. This was because these datasets could be fitted easily without any hidden layer given the convenient set of input features. As the number of layers increased, more epochs were needed to converge the network thus runs for lower epochs did not pass the filter we set earlier on train_loss.

We next looked at the activation functions. There were four choices for the activation function to be applied to the output of all hidden layers: “linear”, “relu”, “sigmoid”, and “tanh”. The linear activation function, was simply passing the input to output (i.e., *f*(*x*) = *x*), while the other three added some nonlinearity to the output of layers, something which is required when classes cannot be fitted properly using only a linear combination of input features. Figure 13E shows the histogram of the runs using each activation function. As expected, all four activation functions performed similarly on the linearly separable gauss dataset. For the “spiral” dataset we saw that only the runs using either of the three nonlinear activation functions converged. However it appeared as if the choice of activation function had little effect on the outcome of the runs on the “circle” and “xor” datasets. Our hypothesis was that those runs were benefiting from the nonlinearity of their input features. So, to verify that, we filtered out all runs which used any input feature other than *X*_1_ and *X*_2_. As shown in Figure 13F, for the gauss dataset all four activation functions still had a similar outcome, but for the other three datasets, only the runs with nonlinear activation functions converged.

**FIGURE 14:**
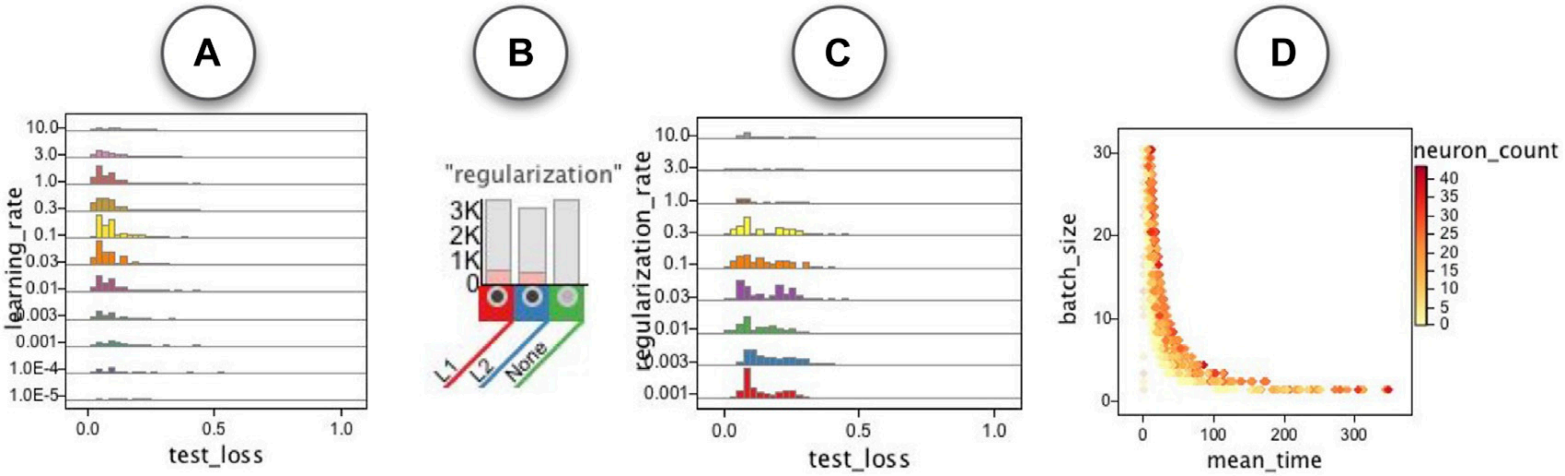
Some patterns common in the runs for all datasets: **(A)** learning rate. **(B,C)** regularization rate for the runs with L1 and L2 regularization. **(D)** Association of mean epoch time with batch size and neuron count.

In addition to the patterns we observed for the runs for each specific dataset, we also observed some patterns common in runs for all datasets. As shown in Figure 14A the most effective values for the learning rate seemed to be in the [0.001, 1.0] range. To study regularization rate, we first selected runs that used either L1 or L2 regularization (Figure 14B) and observed that the most effective values for the regularization rate seemed to be in the [0.001, 0.3] range. We were also interested in the computational cost of the networks. We explored association of different hyperparameters with the average epoch time and noticed the batch size and neuron count to be the two hyperparameters showing the strongest association with the mean epoch time (Figure 14D).

## 6 Conclusion and future work

In this paper we introduced ModEx, a novel general purpose system for visual parameter analysis of computer models. By offering key components of a visual parameter analysis framework including parameter sampling, deriving output summaries and an exploration interface, as well as a flexible API for further extension, ModEx can be employed in a variety of application domains with a reduced development time. We demonstrated the usability and flexibility of our system in three application domains: data mining, machine learning and bioinformatics. However there remains several important limitations that provide opportunities for extending the current work. We would like to address these limitations while we continue evaluating our system on more scenarios in different application domains.

### 6.1 Parameter sampling

Our current implementation only uses random uniform sampling of the parameter space. We did not experiment with other sampling methods as it was not a direct focus of our study. We leave detailed studies of different parameter sampling methods, such as grid sampling, or stratified random sampling, as important future work. Moreover, ModEx does not include the “prediction” component of the conceptual framework for visual parameter space analysis the purpose of which is to predict or estimate model outputs for parameter combinations that have not been sampled.

### 6.2 Analyzing output

Currently we only allow a side-by-side comparison of output images in the exploration interface. However, we believe better comparison methods of images as well as output data tables will improve the effectiveness of analysis. A different type of useful comparison, is the comparison of multiple computer models together. Currently to compare different models together, a user needs to first combine them into a meta-app similar to our approach for the Clustering app. Another missing feature is the ability to group runs with similar output (images or data) to reduce the clutter during exploration and better help with finding patterns or anomalies. Note that in some cases a brute-force grouping by comparing the output values may not work well, for example, when the output are IDs for cluster labels.

### 6.3 Browsing parameters

Our current implementation displays parameters and derived output independent of each other. However in many models, dependent parameters may exist. For example, in our Clustering app, the algorithm parameter is only relevant when the clustering method is set to k-means. The R-app API already allows declaring these dependencies to show parameters only as needed, however this information is not utilized during the exploration. We initially experimented with a hierarchical view of parameters instead of a list, however the resulting UI turned out to be too complex and we opted out to a basic list view until we find a better alternative. Another useful feature when browsing parameters is to be able to sort parameters by some sort of importance metric that measures the significance of the parameters on the output of the models.

### 6.4 Guiding the exploration

In our current exploration interface the user has to manually experiment with different configurations of axis to look for interesting patterns. One area of potential improvement is to utilize scagnostics Wilkinson et al. (2005) to help the user find interesting views of the data.

## Data Availability

The original contributions presented in the study are included in the article/Supplementary Material, further inquiries can be directed to the corresponding authors.
